# Safety and feasibility of a home-based six week resistance training program in juvenile idiopathic arthritis

**DOI:** 10.1186/1546-0096-11-46

**Published:** 2013-12-20

**Authors:** Cameron Van Oort, Susan M Tupper, Alan M Rosenberg, Jonathan P Farthing, Adam D Baxter-Jones

**Affiliations:** 1 College of Kinesiology, University of Saskatchewan, 87 Campus Drive, Saskatoon, SK, S7N 5B2 Canada; 2 Saskatoon Health Region, University of Saskatchewan, 87 Campus Drive, Saskatoon, SK, S7N 5B2 Canada; 3 Department of Pediatrics, College of Medicine, University of Saskatchewan, 87 Campus Drive, Saskatoon, SK, S7N 5B2 Canada

**Keywords:** Juvenile idiopathic arthritis, Pain, Resistance training, Ultrasound, Childhood health assessment questionnaire, Accelerometry

## Abstract

**Background:**

Juvenile idiopathic arthritis (JIA), among the most common chronic diseases of childhood, can be associated with attenuated physical activity levels, reduced fitness, decreased functionality and pain. This pilot study aimed to determine the safety, feasibility and effect of a six week resistance training program in children with JIA.

**Methods:**

Youth (8-18 years) with JIA participated in a home-based resistance training program. Participants reported pain on an electronic diary once a day for one week prior to training, then once a day on non-exercise days and three times a day (before-exercise, after-exercise, and end-of-day) on exercise days for the subsequent six weeks of training. Secondary outcome measures included inflammation (assessed by ultrasound), muscle size (assessed by ultrasound), muscle strength (assessed by dynamometer) and functional ability (assessed by childhood health assessment questionnaire), measured at baseline and post-training. Participants were also instructed to wear an accelerometer one week prior to training to estimate baseline physical activity levels. Statistical analyses included safety (pain changes and any adverse events), feasibility (adherence to program and modifications made to exercises) and effect of program (differences in secondary measures pre and post training). An alpha level of *p* < 0.05 was accepted as significant.

**Results:**

Seven participants completed an average of 12.7 ± 3.4 (range 8-17) exercise sessions out of a possible 18 (70.6%). No adverse events were reported and pain did not increase over the seven weeks. Secondary measures revealed a significant increase in vastus lateralis thickness from pre to post training (*p* < 0.05). End-of-day pain intensity was correlated to end-of-day stiffness, fatigue and mood (*r* = .864, *r* = .581, *r =* -.637, respectively, *p* < 0.001). Pain intensity was also correlated with ratings of perceived exertion of the exercise (*r =* 0.324, *p* < 0.01). Only two children met the recommended 60 minutes of moderate to vigorous physical activity per day.

**Conclusions:**

A six week home-based resistance training program is both safe (absence of pain changes or adverse events over the six weeks) and feasible (comparable adherence rates to other exercise studies involving JIA and individually modifiable) in children with JIA.

## Background

Juvenile idiopathic arthritis (JIA) is the most common class of rheumatic disease affecting children [1]. Sequelae of JIA can include pain, reduced fitness, lower quality of life, decreased functionality and less participation in social activities [2-4].

Children with JIA also demonstrate attenuated levels of physical activity relative to their healthy counterparts [5,6], which further compounds the adverse effects of JIA on musculoskeletal health [6-9]. A key reason for this divergence in physical activity could be the amount of daily pain experienced, as daily pain is predictive of lower physical activity levels and reduced quality of life [10,11].

Chronic inflammatory joint disease is characterized by changes in levels of ions (potassium and hydrogen), bradykinin, prostaglandins, histamine, 5‒hydroxytryptamine, adenosine triphosphate, nitric oxide, cytokines, leukotrienes and growth factors, that infiltrate the synovium to cause inflammatory changes. Inflammatory mediators can act on nociceptors directly to initiate pain, cause the release of additional algogenic substances, or modify the response properties of primary afferent neurons causing sensitization [12]. Sensitization within the nervous system can lead to allodynia, hyperalgesia and/or ectopic nerve firing [13].

Although extensive research into the effects of prolonged resistance training in children with JIA has not been performed, there is potential for positive benefit with this method of training. Attenuated levels of tumor necrosis factor alpha, a pro-inflammatory cytokine, have been measured after an eight week resistance training program in patients with multiple sclerosis [14]. Furthermore, exercise training positively influences circulating levels of pro-inflammatory cytokines and toll-like receptors in various disease populations [15-19]. Therefore, resistance training could decrease inflammation and subsequently decrease pain in addition to improving muscular strength and endurance [17,19,20]. Children with JIA report distance of travel to a supervised exercise program, scheduling conflicts and a lack of time as reasons for not adhering to an exercise program [21,22]. Consequently, clinicians and researchers must explore creative ways to facilitate the acceptability and feasibility of exercise programs. Home-based programs offer the benefits of being affordable while limiting the demands for travel, supervision and schedule coordination [21,22].

The purpose of this pilot study was to examine the safety and feasibility of a home-based, six week resistance training program for children with JIA. Additionally, this study was intended to collect pilot data for future research evaluating resistance training as a component of JIA management.

## Methods

Children between the ages of 8 and 18 years with JIA who attended the Pediatric Rheumatology Clinic, Royal University Hospital (RUH), Saskatoon, Canada were enrolled. Inclusion criteria were diagnosis of any subtype of JIA with either active or inactive disease. Exclusion criteria included an absence of arthritis related pain within the last six months. All procedures were approved by University of Saskatchewan’s Research Ethics Board (Bioethics approval number-12-241) with informed consent and assent attained from each parent and participant, respectively.

Height and weight measurements were performed twice, at baseline and post-exercise visits. The average of two measures was recorded at each time point. Height was measured to 0.1 cm on a wall mounted stadiometer (Holtain Ltd. of Crymych, Dyfed, UK.) and weight was measured to 0.1 kg on a weight scale (Toledo Scale Company of Canada, Windsor, Ont.). Age was measured to 0.1 years (calculated by the number of days after the most recent year and divided by 365.25).

The intervention was a combined body weight (BW) and resistance band (RB) (TheraGear® Canada Ltd., Mission, BC, Canada) training program lasting six weeks. Participants exercised three times per week for approximately 40 minutes each session in a circuit style of training. The program consisted of a five minute warm up, 25 minutes of resistance training and an eight minute cool down. Squats (with RB), lunges (BW), step-ups (BW), planks (BW), combined bicep curl and shoulder press (RB), seated row (RB) and push-ups (BW) were each completed for three sets (refer to Additional file 1 for exercise pictures and descriptions). These exercises were selected to provide training to major muscle groups of the body. The program progressed biweekly in number of repetitions and exercise difficulty. Initial RB selection and method of performing BW exercises was established in a training session (with each exercise demonstrated) where participants were able to complete eight full repetitions of each exercise, but could not complete twelve. During the subsequent training, participants were told to adjust foot placement on the RB or progress in the BW exercises as demonstrated in order to be able to complete the required number of repetitions, but would struggle to do two more. Participants were shown modifications of exercises in the event that certain exercises caused joint pain. Participants also received a video that visually demonstrated and verbally described the circuit so that each session could be followed at home. Parents were present at baseline and post testing and helped with the scheduling and monitoring of exercise session completion (instructed to have at least one day between training sessions), but participated in the exercises only by choice and this was not accounted for. If at any point during the study, participants or their parents felt adverse events were occurring due to the exercise regime, they were asked to report to the research coordinator and rheumatologist.

Pain was reported using a tablet application labeled PinGo© and adapted from previous diaries with demonstrated construct validity [23-25]. Participants answered pain and arthritis based questions once per day at the end of the day (end-of-day) for the week preceding the exercise program to estimate baseline pain. During the training period, pain was reported once daily (end-of-day) on non-resistance training days and three times a day (before-exercise, after-exercise, end-of-day) on resistance training days throughout the six week time course to determine the effects of resistance exercise on pain. Participants were asked to report pain intensity (PI) (using a visual analog scale), pain affect (PA) (using the Facial Affective Scale) [26] and pain interference (PIn) (using a visual analog scale). As well, they reported their perceived stiffness, fatigue and mood. Specific to the after-exercise questionnaire, participants were also instructed to report ratings of perceived exertion (RPE) of the exercise (adapted with permission from the Omni RPE scale [27]) and any adaptations made to the exercises. This was to understand which exercises adversely affected the individual’s (safety) and to evaluate adherence to the program based off the number of after-exercise questionnaires completed (feasibility). All tablets used were Google Android (Google Inc. Mountain View, CA, United States).

Ultrasound (Zonare, L14-5 MHz Linear Wide Array Transducer) was used to assess intra-articular pathology. The largest currently active joint or largest previously active joint (dominant side preferentially selected if two equally sized joints affected) was imaged at baseline and after the training program (within three days of completion). Fluid, synovial hypertrophy and the presence of increased synovial blood flow on power Doppler were observed to determine intra-articular pathology (overall inflammation) and each parameter scored semi-quantitatively on a zero to four scale where zero is not present at all and four is maximally present.

Muscle thickness (cross sectional area) of the vastus lateralis (VL) and the biceps brachii (BB) (chosen due to the nature of the training, minimal subcutaneous fat and previous research on these sites) were measured using B-mode ultrasound and a 12 MHz transducer placed over the muscle belly (LOGIQ e BT08 GE Healthcare Scanner; GE Medical Systems, Milwaukee, Wisconsin, USA). The VL was located by measuring from the top of the greater trochanter to the lateral epicondyle of the femur and taking the 70% mark between those two points, beginning at the proximal end. The BB was located by measuring from the anterior aspect of the acromion process of the scapula to the proximal aspect of the cubital fossa with the arm in a 90 degree flexed position and taking the 70% mark between those two points from the proximal end. Measurements were performed at baseline and after (within three days of completion) the exercise training program. Both measurements were completed four times for each muscle, taking the average of the two closest measurements to determine muscle hypertrophy of the respective sites [28]. For further detail of muscle thickness measurements refer to Chilibeck et al. [29].

Muscle strength of the leg extensors and elbow flexors were assessed before and after the exercise program using the Humac NORM Dynamometer (CSMi of Stoughton MA, USA). Participants were seated with the anatomical axis of rotation for each movement in line with the axis of rotation of the dynamometer. Stabilizations straps were used to prevent body parts from moving that did not surround the anatomical axis of rotation. Three warm-up repetitions were performed to allow the participant to become accustomed to the movement. Four maximal voluntary contractions were subsequently performed at 60 degrees per second (1.05 radians/sec) for each muscle group, with a one minute rest between each repetition. The greatest of those measurements in newton metres was recorded.

Functional ability was assessed using the Childhood Health Assessment Questionnaire (CHAQ) at baseline and after the exercise training program (within three days of completion) [30]. The questionnaire is divided into three portions: disability index, discomfort index (pain), and health status (quality of life). CHAQ is reported on a zero to three scale, with zero meaning the child has full function. Due to the comprehensive and concurrent measure of pain in this study, discomfort index was not used in the analysis.

Baseline physical activity was objectively measured in participants using accelerometers over a seven day period, and in conjunction with end-of-day pain measurement for the week preceding initiation of the exercise program. Participants wore the ActiGraph GT3X + accelerometer (Actigraph© of Pensacola, FL, United States) around their waist during waking hours with epoch length set at three seconds. The ActiLife version 6.4.5 software (Actigraph© of Pensacola, FL, United States) was used to initialize the devices, as well as download the collected data from the accelerometers. Participants were also given a log to note when, the duration and reasons they removed the accelerometer.

Finally, a closing questionnaire asked the participants their thoughts about the exercise program. These included enjoyment of the program, potential modifications and its effect on their overall quality of life.

Adherence to the program was analyzed by determining the number of after-exercise questionnaires completed. All other data were entered and analyzed using SPSS software version 20© (IBM Corporation, Armonk, New York). End-of-day PI, PA and PIn scores were averaged for each participant over the initial baseline week, first two weeks of training, middle two weeks and final two weeks. Repeated measures analysis of variance (ANOVA) was performed to test differences between those four time points for the whole group. PI change scores from before to after each exercise session were computed by subtracting the PI before-exercise from the PI after-exercise. Each occasion was then used to determine the number that decreased below 8.2 units and the number that increased above 19.0 units (clinically relevant changes in PI for children with rheumatologic disease) [31]. Dependent t-tests were run for each individual between before-exercise and after-exercise PI and PA scores. Pearson’s correlation was used to assess the bivariate relationship between before-exercise PI, after-exercise PI, and RPE of each participant and pooled based on the number of exercise sessions completed by each. Pearson’s correlation also assessed the bivariate correlation between end-of-day PI and end-of-day stiffness, fatigue and mood for each individual based on the number of end-of-day questionnaires completed. Changes in inflammatory variables, muscle variables and functional ability from pre to and post exercise program were analyzed using paired-samples dependent t-tests. Significance was considered acceptable at an alpha level of p < 0.05. G*Power 3.1.7 (G*Power© of Dusseldorf, Germany) was used to calculate the necessary sample size for a definitive study to achieve a power of 80%.

## Results

Seven participants (four females, three males) participated in this study out of an approached 20. Those who decided not to participate either expressed no interest, lived too far away or did not meet the inclusion criteria. Three participants had seronegative polyarticular JIA, three had oligoarticular JIA and one had enthesitis related arthritis. Before starting the exercise program four participants had active disease and three inactive disease. Upon completion of the program one participant had active disease and six inactive disease (one began taking etanercept during the study). Table 1 displays the participant demographics with results reported as means ± standard deviation. On average, female height-for-age fell just below the 50th centile and males were between the 15th and 25th centile, compared to World Health Organization growth reference charts. The female and male BMIs placed them between the 85th and 95th centiles and 50th and 75th centiles, respectively.

**Table 1:** Characteristics of participants from before to after the six week exercise program

**Variable**	**Before**	**After**
Age (yrs)-females	14.9 ± 3.0	15.0 ± 3.3
Age (yrs)-males	13.5 ± 2.2	13.6 ± 2.4
Height (cm)-females (n = 4)	161.6 ± 11.6	161.2 ± 10.4
Height (cm)-males (n = 3)	153.9 ± 17.2	53.9 ± 17.2
Height (cm)-combined (n = 7)	158.3 ± 13.5	158.6 ± 13.0
Weight (kg)-females (n = 4)	62.8 ± 19.4	62.8 ± 19.8
Weight (kg)-males (n = 3)	42.8 ± 15.4	43.0 ± 16.1
Weight (kg)-combined (n = 7)	54.2 ± 19.5	54.3 ± 19.8
BMI (kg/m^2^)-females (n = 4)	24.1	24.7
BMI (kg/m^2^)-males (n = 3)	18.7	18.2

No significant differences existed between before and after training values.

The average number of exercise sessions completed (based on the number of after-exercise questionnaires completed) was 12.7 ± 3.4 out of a possible 18 (70.6%). Zero participants completed all 18 exercise sessions; the highest number was 17 completed by two participants. The average number of before exercise pain questionnaires completed was 12.7 ± 3.4. The average number of end-of-day pain questionnaires completed was 36.0 ± 10.1 out of a possible 49. The highest number of end-of-day pain questionnaires completed was 47 by one of the participants. No adverse events were reported from any participant by way of either by contacting any of the researchers or reporting adverse events via notes on the tablet. As well, no dropouts occurred in this study.

Repeated measures ANOVA of averaged PI, PA and PIn scores for the baseline week, the first two weeks of training, middle two weeks of training, and final two weeks of training revealed no significant differences.

The average change in PI from before-exercise to after-exercise in a single exercise bout was an increase of 3.0 ± 11.8 units (out of possible 100 unit change). The number of occurrences (each occurrence is a single exercise session with a respective before and after-exercise questionnaire) that had a clinically significant decrease in PI of at least 8.2 units was seven with a mean decrease of 19.9 ± 9.1 units. The number of occurrences that had a clinically significant increase in PI of at least 19.0 units was seven with a mean increase of 26.7 ± 6.6 units. The total number of occurrences that did not increase or decrease in PI to a clinically significant amount was 60 out of 74. A decrease in pain to any extent occurred in 17 out of the 74 total occurrences and an increase in pain occurred in 31 out of the 74 cases.

When PI and affect were analyzed within individuals from before to after-exercise for each session, no statistically significant differences existed for either variable. With all participants aggregated into the analysis, no significant differences existed again for either of the variables.

Table 2 displays the ages and disease subtype, the number of sessions completed, the range of scores of PI before exercise, the range of scores of PI after-exercise and the range of RPE scores for each participant. Aggregated before-exercise PI was positively correlated to after-exercise PI (p < 0.001). Aggregated before-exercise PI was positively to RPE of the exercise (p < 0.01). Aggregated after-exercise PI was positively to RPE of the exercise (p < 0.01).

**Table 2:** Pain intensity before-exercise, pain intensity after-exercise and RPE of participants individually

**Participant**	**Age/disease subtype**	**Pain intensity before mean (range)**	**Pain intensity after mean (range)**	**RPE mean (range)**	**# of training sessions completed**
1	10.2/SNP	12.1 ± 26.7 (0-83)	12.9 ± 31.7 (0-92)	7.08 ± 2.4 (2-10)	12
2	11.8/SNP	7.9 ± 8.8 (0-23)	11.5 ± 15.3 (0-50)	3.7 ± 0.8 (2-5)	10
3	15.8/OA	46.9 ± 23.7 (13-88)	52.7 ± 27.1 (0-100)	7.1 ± 0.6 (6-8)	17
4	16.3/ER	MD	MD	MD	MD
5	16.8/OA	23.6 ± 17.1 (0-50)	20.4 ± 25.4 (0-68)	6.4 ± 0.5 (6-7)	8
6	12.9/SNP	27.8 ± 32.2 (0-81)	25.4 ± 32.5 (0-74)	4.5 ± 1.3 (3-7)	17
7	17.0/OA	63.8 ± 13.8 (34-87)	68.69 ± 10.1 (56-92)	6.4 ± 0.5 (6-7)	12

*Abbreviations*: SNP seronegative polyarticular, OA oligoarticular, ER enthesitis related, MD Missing data due to tablet malfunction.

Bivariate correlations of each of the following variables, pooled and individually are shown in Table 3: Pooled bivariate correlations revealed end-of-day PI was significantly positively correlated to end-of-day stiffness (*p* < 0.001) and fatigue (*p* < 0.001), while negatively correlated to mood (*p* < 0.001). Individually, five of six, two of six, and three of six (only six participants contributed to the pain measure data) participants end-of-day PI significantly correlated to their end-of-day stiffness, fatigue and mood, respectively (*p* < 0.05). PI before-exercise was significantly correlated to PI after-exercise for five of six participants (*p* < 0.01) and overall (*p* < 0.001). PI before-exercise and PI after-exercise were both significantly correlated to RPE for one of six participant’s (*p* < 0.01) and overall (*p* < 0.001).

**Table 3:** Individual and pooled correlations between pain intensity and other self-reported variables

	**R range**	**No. of participants with a significant correlation (P < 0.05)**	**R pooled (across all data points from all participants)**	**Significance of R pooled**
End-of-day PI and stiffness	.221 to .911	5 of 6	.864	*p* < 0.001
End-of-day PI and fatigue	.120 to .833	2 of 6	.581	*p* < 0.001
End-of-day PI and mood	-.836 to .244	3 of 6	-.637	*p* < 0.001
PI before-exercise and PI after-exercise	.748 to .980	5 of 6	.912	*p* < 0.001
PI before-exercise and RPE	-.524 to .785	1 of 6	.327	*p* < 0.001
PI after-exercise and RPE	-.410 to .867	1 of 6	.324	*p* < 0.01

Note: Only 6 out of 7 participants contributed data for the correlation analyses.

*Abbreviations*: PI pain intensity, RPE ratings of perceived exertion.

Fluid, synovial thickening and synovial blood flow were not significantly different from pre to post-resistance training. Muscle thickness of the VL increased from before to after the program (*p* = 0.017). All other muscle parameters did not significantly change for pre- to post-training. Disability index and health status also did not significantly differ. These variables are displayed in Table 4 along with a required sample size for definitive research.

**Table 4:** Measures of pre and post six weeks resistance training program and required sample size for 80% power

**Measures of pre and post 6 weeks training**	**Pre training**	**Post training**	**Required sample size for 80% power**
Inflammation (Likert scale) (n = 7)	1.0 ± 1.2	0.1 ± 0.4	29
Fluid (Likert scale) (n = 7)	1.0 ± 1.2	0.3 ± 0.5	26
Synovial thickening (Likert scale) (n = 7)	0.6 ± 1.1	0.6 ±0.8	Effect size not large enough
Synovitis (Likert scale) (n = 7)	0.6 ± 0.8	0.1 ± 0.4	46
Vastus lateralis thickness (cm) (n = 7)	3.0 ± 0.6	3.2 ± 0.7*	7
Biceps brachii thickness (cm) (n = 5)	2.6 ± 0.4	2.9 ± 0.5	8
Leg extension strength (n.m) (n = 7)	111.6 ± 42.9	105.0 ± 50.5	22
Elbow flexion strength (n.m) (n = 7)	28.3 ± 11.3	30.1 ± 18.0	275
Disability index from CHAQ (0 to 3 continuous scale) (n = 7)	0.4 ± 0.7	0.2 ± 0.3	47
Health status from CHAQ (Likert scale) (n = 7)	0.4 ± 0.6	0.2 ± 0.2	131

*Significant difference from pre training.

cm = centimetres.

n.m = newton metres.

**Figure 1:**
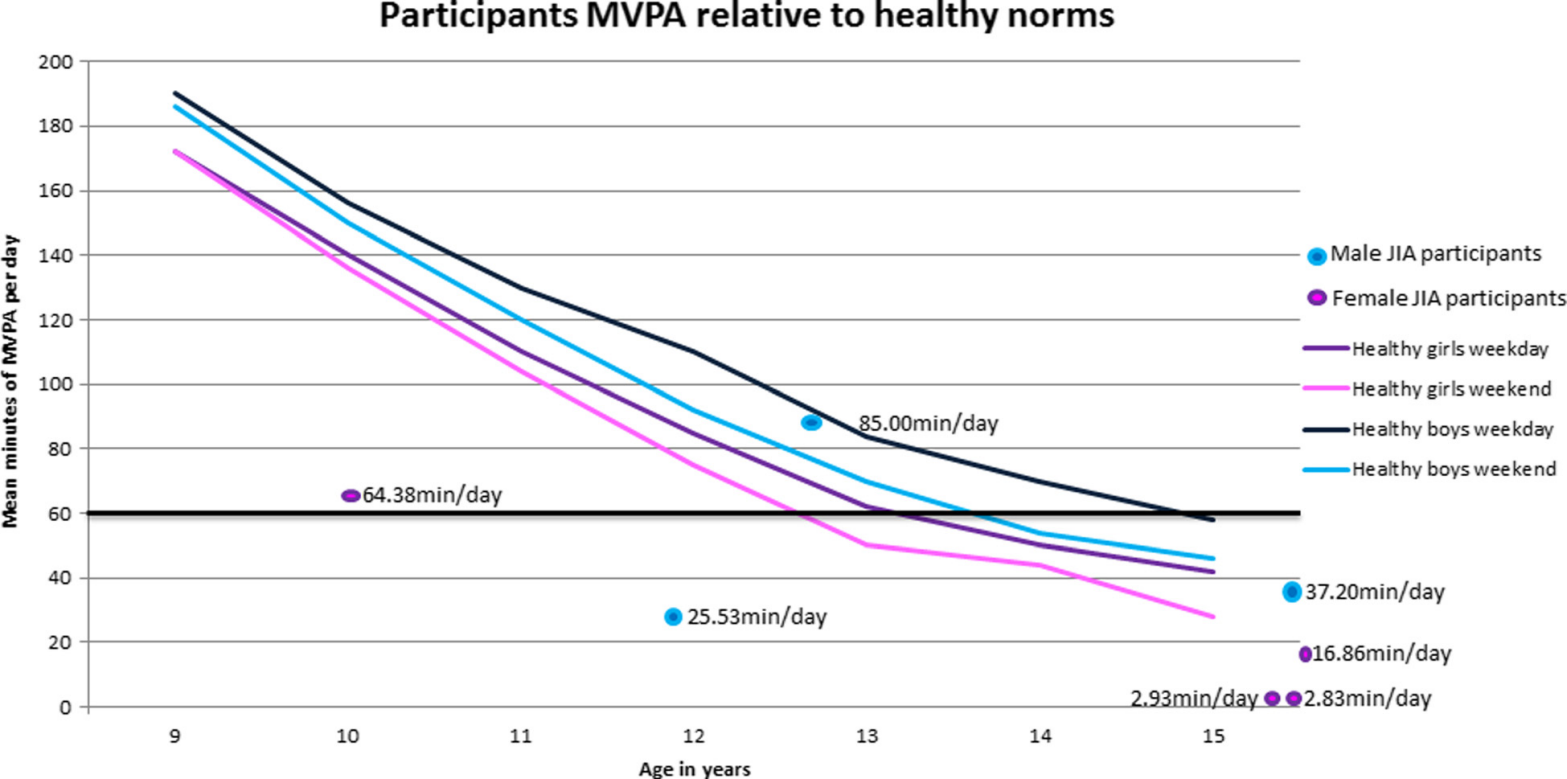
**Baseline moderate to vigorous physical activity (MVPA) in children with JIA relative to healthy peers.** Reproduced from Nader et al (30) study of healthy children from ages nine to 15. The solid lines depict average minutes of MVPA for both healthy males and females at their respective ages, as well as for weekend days and weekdays. Positioned on this graph are also the seven children with JIA (four females represented by purple/pink dots and three males represent by blue dots) from this study and the individual average MVPA over seven days.

Baseline physical activity is depicted in Figure 1, with a comparison of each participant to the recommended 60 minutes of moderate to vigorous physical activity (MVPA) per day (bold black line). Reference lines of physical activity for healthy children between the ages of 9 and 15 years are also shown in the figure from previous research [32]. One female and male participant met the recommended 60 minutes of MVPA per day and only one male fell within the averages of healthy age-matched children. All other participants were under the recommended 60 minutes of MVPA for their age and were also less than their age-matched peer averages.

All participants reported that they felt the exercise program was enjoyable. Two participants reported that the exercise program became more challenging as the weeks progressed due to the greater number of repetitions required, but that they felt it was a positive aspect of the program to increase in challenge over time. One participant felt that a baseline level of athleticism was necessary to complete the exercise program. One participant felt it to be difficult at first and that their muscles were sore the following day, but that as the weeks progressed less muscle soreness occurred. A competitive bowler in the study reported that the exercise program improved endurance in bowling competitions. Participants also felt that the exercise program would have enhanced with background music. One participant who had left elbow pain for two out of the 14 completed exercise sessions decided use only the right arm for exercises of the upper body.

## Discussion

The primary purpose of this study was to examine the safety and feasibility of a home-based resistance training program in children with JIA. A duration of six weeks was chosen for this pilot research as previous research has demonstrated as little as four weeks of training to be effective in children and adolescents [33]. The results presented suggest the program is safe, feasible and acceptable as indicated by the absence of adverse events from the study participants (safety), overall non-significant increases in pain (safety), comparable participation rates (feasibility) and qualitative feedback (acceptability). Some of the participants reported modifications of an exercise or exercises in order to complete the program, demonstrating ability to tailor the program to individual demands.

For the purposes of this study and this population, an 8.2 unit decrease or a 19.0 unit increase in PI from before-exercise to after-exercise was considered clinically significant. This is based on the work of Dhanani et al. [31] who examined correlations between changes in recalled summary scores of pain reported by 553 children with mixed rheumatic diseases who experienced chronic pain. At two consecutive clinical visits, self-reported perceptions of change in overall health status were measured along with changes in PI to determine the amount of change in PI associated with a decrease or increase in health status. Various change scores have been used to signify clinically meaningful changes in pain scores, such as a 1 or 2 unit change on the 11 point numeric rating scale or 10-20% change [34-36]; however, the majority of these studies report meaningful changes in adults with acute pain. The Dhanani et al. [31] study reports meaningful change in pain for a comparable population (pediatric rheumatic disease) and was therefore thought to be most applicable. In the present study seven of the PI change scores (exercise sessions) decreased greater than 8.2 units and seven increased greater than 19.0 units from before-exercise to after-exercise sessions. As well, 60 of the current observations did not show a clinically significant change in PI (the most widely used measure of pain) from before-exercise to after-exercise. Furthermore, end-of-day pain did not increase over the training program. This demonstrates that resistance training did not significantly increase pain either acutely after exercise or longitudinally over the duration of six week resistance training program in children with JIA. It should be noted, however, that other meaningful changes in pain may have occurred that were not captured by this study, such as delayed onset muscle soreness, or increases in pain that occurred following the after-exercise survey that dissipated prior to the end-of-day survey.

Participants were able to complete 70.6% of prescribed exercise sessions. Takken et al. [37] conducted a pilot study on the effects of a group-based aerobic aquatic training program in children with JIA. The adherence rate was 85% with participants missing the training sessions due to disease, lack of transportation or conflicting social activities. Singh-Grewal et al. [38] reported adherence rates of 56% in their high intensity aerobic exercise experimental group compared to 78% in their control group. A pilot study conducted by Singh-Grewal et al. [39] saw adherence rates of 63% for a supervised circuit training program in children with JIA. Finally, a study by Sandstedt et al. [40] had participants perform a 12 week exercise program including jump rope, free weight and body weight exercises, with an adherence rate of 70% of the expected value, with five out of 28 participants in the training group dropping out. The current study demonstrated similar rates of adherence, and was unique in that it was home-based indicating less social influence and an ability to adapt the program to individual schedules. Therefore home-based resistance exercise appears to be feasible for children with JIA and could be a cost-effective form of treatment for both the patient and healthcare system.

The current study also suggests that home-based resistance training could potentially lead to muscle hypertrophy. The thickness of the VL significantly increased by 6% after the six weeks of exercise training (refer to Table 4). Previous research has demonstrated conflicting results regarding physiological adaptations to exercise training in this population [21,22,38,40]; However, this is the first study of its kind to analyze muscle thickness in children with JIA after a strength training program; to adjust for changes due to normal growth and development would require a randomized control trials to determine definitive effects.

Pain and stiffness and pain and fatigue displayed correlations for some individuals and overall. Previous research suggests that pain, stiffness and fatigue are all significant predictors of reduced participation in school and social activities in children with JIA [41]. As well, mood has been shown a significant predictor of pain, stiffness and fatigue in children with JIA [42]. The current study also demonstrated negative correlations between mood and pain. The results also suggest PI and RPE to be correlated overall and within some individuals in this study. There may therefore be potential that by reducing pain one would be more inclined to be physically active. Future research should aim to determine whether altering one of these variables impacts other variables, such that if morning stiffness or mood were improved in this population, potentially pain reduction could occur.

The results of this study are unique as it is the first to analyze the safety, feasibility and pilot results of effects of a home-based resistance training program on pain, inflammation, muscular fitness and functional ability in children with JIA. However, the conclusions are limited by several factors. First, the sample size of this study was such that definitive results cannot be attained. Furthermore, the participants in this study did not represent all disease subtypes of JIA and further research is needed to understand the generalizability to the broader clinical population. This would include patients with systemic JIA and rheumatoid factor positive polyarticular JIA. Secondly, the duration of the exercise program could be extended with mid time point measures of secondary variables to determine longitudinal changes. Finally, although this study used clinically significant changes based on those reported by Dhanani et al. [31], these change scores may not be meaningful changes in pain for all youth with JIA. Individually determined change scores may produce different results [34-36].

## Conclusion

In conclusion, despite the widespread understanding that physical activity, including exercises targeting muscular strength and fitness, is a necessary component for optimal growth, there has been little research on the feasibility, safety and effectiveness of resistance training in JIA. The majority of studies have examined the effects of aerobic training programs on fitness and function in youth with JIA. The present demonstrated children with JIA were able to safely participate in the program without significantly exacerbating their pain. Future research is necessary with larger sample sizes and potentially longer duration resistance training programs to definitively determine the effects of resistance training in children with JIA.

### Consent

Written informed consent was obtained for the publication of this report and any accompanying images.

## Abbreviations

JIA: Juvenile idiopathic arthritis; PI: Pain intensity; PA: Pain affect; PIn: Pain interference; RPE: Rating of perceived exertion; ANOVA: Analysis of variance; VL: Vastus lateralis; BB: Biceps brachii.

## Competing interests

The authors declared that they have no competing interests.

## Authors’ contributions

CV carried out the study design and conception, data collection, data analysis, data interpretation, and writing of the manuscript. ST carried out study design and conception, data analysis, data interpretation, and revising of the manuscript. AR carried out study design and conception, data collection, and revising of the manuscript. JF carried out study design and conception and revising of the manuscript. ABJ carried out study design and conception and revising of the manuscript. All authors read and approved the final manuscript.

## Supplementary Material

Additional file 1 Exercises of the home-based resistance training program. Click here for file
