# Supplementary material for: Safety and feasibility of a home-based six week resistance training program in juvenile idiopathic arthritis
Source: Pediatr Rheumatol Online J. 2013 Dec 20;11:46. doi: 10.1186/1546-0096-11-46 (PMC3878188; doi:10.1186/1546-0096-11-46)
Supplement: Additional file 1 — Exercises of the home-based resistance training program. [file 1546-0096-11-46-S1.docx]

Additional file 1: Exercises of the home-based resistance training program

Squat-RB under the sole of each foot and the handles in the hands while resting on the shoulders. Ideally for this exercise the participant went into 90 degrees of flexion at the knee, but if it was not possible then they were instructed to go to a depth that they were capable of and try to progress to 90 degrees. Participants were also instructed to progress the resistance by widening stance on the RB, bringing the RB up higher, or crossing the RB.

 Lunges- The knee opposite to the lunging leg was to come within six inches of the floor on the downward portion (eight repetitions for each leg). Again, if the participant was not capable of this they were able to go to a depth they were capable of. The first two weeks were stationary, followed by the middle two weeks of returning to feet together, and finally walking forward with lunges in the last two weeks.

 Step ups- The height of the chair or stair was up to the participant, but meant to be at a degree of difficulty they felt possible while staying in a joint pain free range. They were asked to progress the height of the chair or stair as the weeks progressed and the exercise progressed from a standard step up to eventually coming up on the toes.

 Plank- The participant’s forearms and toes were in contact with the ground and their body was held rigid in a straight line. If this was not comfortable, the individual was instructed to place a pillow(s) under their forearms or drop to their knees to attenuate pressure on the forearms. They were asked to progress to holding the exercise for a maximum of 30 seconds, and to progress to being up on their toes with their feet as close together as possible.

 Bicep curl with shoulder press- The hands were in supination and the individual bent at the elbow starting from full extension in order to curl the RB and then press the handles of the resistance band up performing a shoulder press. Participants were instructed to progress the exercise by widening stance on the RB.

 Seated rows-The individual was sitting with the resistance band fastened around a stable object and they were pulling the handles of the band toward their chest. The degree of difficulty depended on how far they chose to sit away from the stable object, and therefore the exercise was progressed by increasing the distance from the object.

 Push-ups- The children performed push-ups either from the knees or toes, or standing leaning toward a wall. The exercise progressed with participants striving to perform the push-ups from their toes and with their elbows tucked close toward their body and their feet together.
